# Species-Specificity in Thermopreference and CO_2_-Gated Heat-Seeking in *Culex* Mosquitoes

**DOI:** 10.3390/insects13010092

**Published:** 2022-01-14

**Authors:** Joanna M. Reinhold, Karthikeyan Chandrasegaran, Helen Oker, José E. Crespo, Clément Vinauger, Chloé Lahondère

**Affiliations:** 1 Department of Biochemistry, Virginia Polytechnic Institute and State University, Blacksburg, VA 24061, USA; reinjm0@vt.edu (J.M.R.); karthikeyan@vt.edu (K.C.); hmoker@vt.edu (H.O.); vinauger@vt.edu (C.V.); 2 Laboratorio de Entomología Experimental—Grupo de Ecología Térmica en Insectos (GETI), Instituto de Ecología, Genética y Evolución, CONICET—Universidad de Buenos Aires, Buenos Aires C1428EGA, Argentina; crespo@ege.fcen.uba.ar; 3 The Fralin Life Science Institute, Virginia Polytechnic Institute and State University, Blacksburg, VA 24061, USA; 4 Center of Emerging, Zoonotic and Arthropod-borne Pathogens, Virginia Polytechnic Institute and State University, Blacksburg, VA 24061, USA; 5 The Global Change Center, Virginia Polytechnic Institute and State University, Blacksburg, VA 24061, USA; 6 Department of Entomology, Virginia Polytechnic Institute and State University, Blacksburg, VA 24061, USA

**Keywords:** *Culex territans*, *Culex quinquefasciatus*, *Culex tarsalis*, mosquito thermal biology, disease vector

## Abstract

**Simple Summary:**

Mosquitoes are cold-blooded insects whose body temperature and metabolism are largely affected by environmental temperature. These blood-sucking insects use heat emanating from their potential hosts to locate them for feeding, which is how they spread deadly diseases. They also use other cues, including exhaled carbon dioxide and other body odors emitted by the hosts. Interestingly, every species displays specific preferences for a range of ambient temperatures and blood hosts, which includes both warm- and cold-blooded animals. To better understand the role of heat in these contexts, we studied female mosquitoes of three species that differ in their location of origin and in their host preference: *Culex territans*, *Cx. tarsalis*, and *Cx. quinquefasciatus*. We analyzed their preference towards specific ambient temperatures and quantified their heat-seeking behaviors in the presence of CO_2_ at different concentrations. We found contrasting differences between these species, which reflects their native habitat and their host preference.

**Abstract:**

Combining thermopreference (T*_p_*) and CO_2_-gated heat-seeking assays, we studied the thermal *preferendum* and response to thermal cues in three *Culex* mosquito species exhibiting differences in native habitat and host preference (e.g., biting cold and/or warm-blooded animals). Results show that these species differ in both T*_p_* and heat-seeking behavior. In particular, we found that *Culex territans*, which feed primarily on cold-blood hosts, did not respond to heat during heat-seeking assays, regardless of the CO_2_ concentration, but exhibited an intermediate T*_p_* during resting. In contrast, *Cx. quinquefasciatus*, which feeds on warm blooded hosts, sought the coolest locations on a thermal gradient and responded only moderately to thermal stimuli when paired with CO_2_ at higher concentrations. The third species, *Cx. tarsalis*, which has been shown to feed on a wide range of hosts, responded to heat when paired with high CO_2_ levels and exhibited a high T*_p_*. This study provides the first insights into the role of heat and CO_2_ in the host seeking behavior of three disease vectors in the *Culex* genus and highlights differences in preferred resting temperatures.

## 1. Introduction

Temperature is an important abiotic factor for all living organisms, especially affecting poikilotherms such as insects, whose body temperature is heavily dependent on environmental temperature. While some insects have developed physiological, behavioral, and morphological strategies of thermoregulation, the primary one for staying within the optimal temperature range consists of moving towards a safe or beneficial temperature to avoid cold or heat stress [1,2]. Consequently, each species shows a thermopreference (T*_p_*), i.e., a preferred range of temperatures based on its physiological needs [2,3,4,5,6]. In mosquitoes, the environmental temperature dictates their distribution, affects life-history traits (e.g., longevity, diapause, length of time spent in different life stages, etc.), and behavioral activity (e.g., flight, rest, feeding, and oviposition) [7,8,9,10]. Furthermore, temperature and heat are critical to hematophagous species such as mosquitoes because, besides affecting their physiology and behavior, thermal cues enable mosquitoes to identify and navigate towards warm-blooded vertebrate hosts for blood feeding. Specifically, mosquitoes rely on differences between environmental and host temperatures to sense and locate hosts via heat-seeking, particularly for landing orientation [11,12,13,14,15,16]. However, it is worth noting that mosquitoes feed on a wide variety of hosts, with some species specializing in feeding on endotherms that vary in body temperatures (e.g., birds: 38–42 °C, humans: 37 °C [17,18,19]), and others specializing on ectotherms, whose body temperature is dependent on environmental temperature. Consequently, mosquitoes feeding on cold-blooded animals do not rely on the host thermal signature to locate them and the mechanisms they employ to seek hosts remains unexplored. Thus, the association between mosquitoes’ preference for a range of temperatures, i.e., thermopreference, and their ability to detect and locate heat sources, i.e., thermo-sensation, is species-specific. While geographic distribution and abundances of mosquitoes correlate with the range of temperature they can tolerate, the species-specific thermal *preferendum* remains largely understudied [20]. Despite heat sensing being a key modality mediating mosquito host-seeking and feeding behaviors with direct consequences on pathogen transmission, it remains understudied in mosquitoes with the exception of some *Aedes* spp. [11,12,14,20]. Addressing these knowledge gaps, this study experimentally characterizes the thermopreference and thermosensation in *Culex* mosquitoes that exploit different ecological niches and display preference towards a range of hosts that greatly vary in body temperature. Mosquito species (Diptera: Culicidae, tribe Culicini and *Culex* genus) *Culex quinquefasciatus* (Say), *Culex tarsalis* (Coquillet) and *Culex territans* (Walker) have overlapping geographic distribution, with *Cx. tarsalis* and *Cx. territans* found primarily in temperate climates and *Cx. quinquefasciatus* in subtropical climates [21,22]. All three species are nocturnal ([unpublished data, [10,23]). *Culex quinquefasciatus* and *Cx. tarsalis* females feed primarily on birds and humans, allowing for virus transmission between these two hosts [24]. However, *Cx. tarsalis* is considered an opportunistic feeder and can also target cold-blooded hosts, including reptiles and amphibians, primarily snakes [25,26,27,28]. *Culex territans* feed on amphibians, reptiles, and occasionally on birds and small mammals [29,30]. These three *Culex* species vector pathogens, including encephalopathic viruses, such as West Nile virus (WNV) and western equine encephalitis (WEEV) [31,32,33,34,35] and parasites, such as avian *Plasmodium*, anuran and reptilian *Hepatozoon,* and avian and anuran *Trypanosoma* [36,37,38,39,40]. The differences in the host preference of these disease vectors show that heat may serve as an important modality for host-seeking in some species, such as *Cx. quinquefasciatus*, but not necessarily for others, such as *Cx. territans*, and may be facultatively used by species such as *Cx. tarsalis*. These host choices may also reflect other aspects of the mosquito life, including thermopreference at rest. However, the perception, preference, and utilization of thermal cues in these species remain unknown. Based on their ecology (e.g., geographic distribution) and biology (e.g., host preference), we hypothesized that each of these three *Culex* species has a distinct preferred temperature range during resting that reflects conditions in their natural habitat. Specifically, we hypothesized that *Cx. quinquefasciatus* and *Cx. tarsalis* show a distinct thermopreference at rest, while *Cx. territans* may be less selective for ambient temperatures. In parallel, we hypothesized that *Cx. quinquefasciatus* and *Cx. tarsalis* show a preference for landing on warmer objects in the presence of carbon dioxide (CO_2_) while *Cx. territans* have little to no preference for heat in the presence or absence of CO_2_. In order to test these hypotheses, we performed thermal gradient assays to quantify the resting temperature preference (T*_p_*) and conducted free-flight heat-seeking assays in the presence and absence of CO_2_ at various host concentrations for the three mosquito species.

## 2. Materials and Methods

### 2.1. Insect Rearing

*Cx. quinquefasciatus* (JHB strain, BEI Resources (NR-43025)) and *Cx. tarsalis* (YOLO strain, BEI Resources (NR-43026)) were reared from eggs hatched in larval trays (Bioquip Industries, Rancho Dominguez, CA, USA) and fed fish food (Hikari First Bites powdered fish food, Kyorin Food Industries, Kansai City, Japan) until they were collected at pupation. *Cx. territans* were collected as larvae at Mountain Lake Biological Station, Pembroke, VA, USA. The larvae were placed into larval trays and fed fish food as previously described until pupation. For each species, pupae of the same age were grouped in emergence funnel containers (Bioquip Industries, Rancho Dominguez, CA, USA), which were moved to a light box (opaque gray box fitted with an internal light source controlled by a timer) within the first two days after collection in order to entrain the adults on a 12 h/12 h light/dark cycle. All experiments were performed in the first 2 h of mosquitoes’ peak activity, i.e., the first 2 h after the offset of the lights. All three species were maintained in climatic chambers (Percival) at 24–26 °C, 70% RH and provided with a 10% sucrose solution ad libitum and starved 24 h before the experiments occurred. Six to ten-day-old mated females were used for both the thermopreference and CO_2_-gated heat-seeking experiments.

### 2.2. Thermopreference Assays

**Figure 1:**
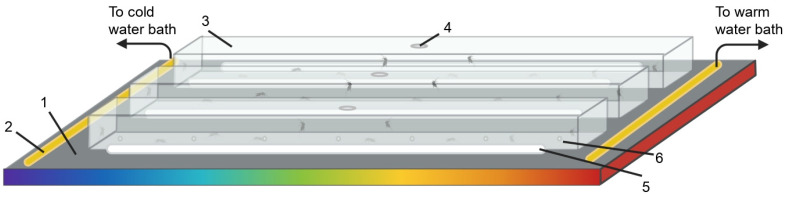
Schematic of the thermal gradient experimental setup. A temperature gradient was created on an aluminum plate (1) using a cold and a warm water bath on either side, connected with copper tubing (2). The mosquitoes were released into acrylic covers (3) through a hole in the top with a fitted acrylic plug (4). Each cover had moist wipe towels (5) and holes drilled on each side to minimize humidity build up (6). The acrylic enclosures are open on their bottom side to allow direct contact of mosquitoes with the aluminum plate.

The thermal gradient was adapted from the devices used in Ritchie et al. [41] and Verhulst et al. [20], consisting of an aluminum plate (86 × 25 × 3 cm, 6061 general purpose aluminum) with custom made acrylic covers (67 × 5 × 2.5 cm; Figure 1). Two temperature gradients (low: 14–34 °C or high: 27–47 °C) across the aluminum plate were created by two waterbaths (F500 Compact Recirculating Cooler, Julabo C-B17 Corio Open Heating Bath Circulator, Julabo, Seelbach, Germany) circulating water through both ends of the plate via copper piping embedded in the aluminum plate. The linearity of the temperature gradient for the surface of the aluminum plate was quantified and verified by placing iButtons across the whole gradient (N = 33; DS1923-F5# Hygrochron Temperature and Humidity, Embedded Data Systems, Lawrenceburg, KY, USA) to determine exact temperature ranges for both experimental conditions. Both the calibration and experiments were conducted 30–45 min after the water baths were turned on to allow for the gradient to be established and stable. In addition to measuring temperature on the plate surface, we inserted thermocouples (Proster Digital Two K-Type Thermocouple Temperature Thermometer) at regular intervals in the acrylic covers to measure the temperature above the plate (i.e., air temperature) to control for a vertical thermal gradient and to obtain more precise T*_p_* data depending on the landing position of the mosquito (i.e., either directly on the plate surface or on the side of the cover). No mosquitoes landed on the top portion of the cover. Moist rolled wipes (Kimwipe, CAT# 34120) were placed on each side of the acrylic cover to minimize the establishment of humidity gradients across the length of the thermal gradient. Humidity gradients created by the moist rollers were also quantified using i-buttons as mentioned above (low temperature gradient: 88–61% RH, average = 77%; high temperature gradient: 72–45% RH, average = 61%). Ten mosquitoes were released through an opening located in the middle of each acrylic cover and were allowed to adjust for 5 min before the experiment started. Up to four assays were conducted in parallel. After 30 min, a photograph of the thermal gradient was taken, and the position of the mosquitoes was reported to the calibration curve corresponding to each experiment to determine T*_p_*. The assays were conducted in a darkened room within one hour from the onset of the scotophase, i.e., mosquitoes’ subjective nighttime. Gloves were worn during the handling of the mosquitoes and equipment to minimize the risk of contamination with human odors.

### 2.3. CO_2_-Gated Heat Seeking Assays

**Figure 2:**
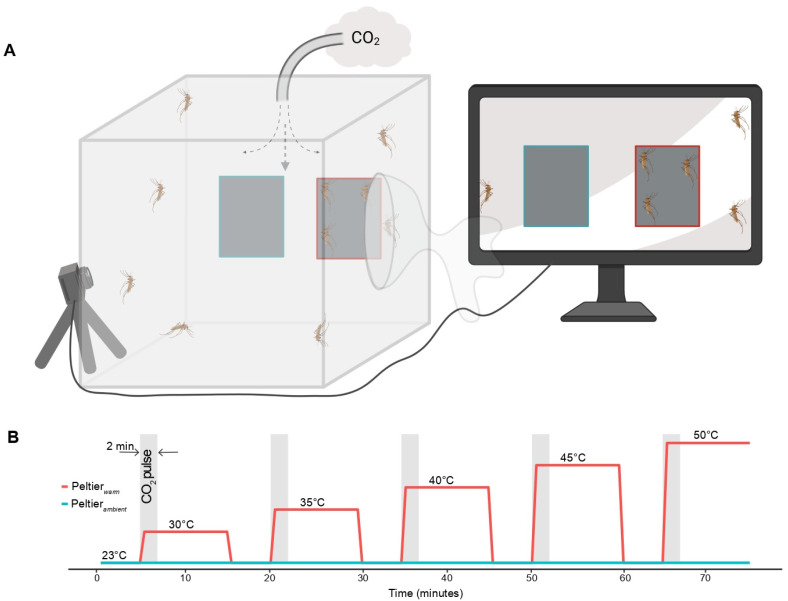
(**A**). Schematic of the heat-seeking experimental setup. Mosquitoes were released into a meshed cage with two Peltier elements directly applied to one side: one maintained at ambient temperature (23 °C, outlined in blue), and one set up to warm up as described in (**B**). A camera recorded the landing activity on the Peltier elements. Through the top of the cage, a tube delivered a constant flow of humidified air to which pulses of CO_2_ were added as described in (**B**). Schematic of ramping temperature steps of the “warm” Peltier element. The warm Peltier element was brought to ambient temperature (2 min) between each step (8 min), which increased the temperature by 5 degrees. Each step began with a 2-min pulse of CO_2_ (in gray).

The heat and CO_2_ seeking assay was performed based on methods adapted from previous studies [11,12]. The setup consisted of a mosquito rearing cage (Rearing and Observation Cage, 12” cube, Bioquip) with two Peltier elements (6 × 4 cm surface area; 12 V 5 A, Peltier Thermo-Electric Cooler Module and Heatsink Assembly, Part # 1335, Adafruit, New Your, NY, USA) equidistantly placed against the mesh lining one of the vertical walls of the cage. A 2 × 2 cm black square printed on standard printer paper (bright white, letter size; Gemini/Liberty paper, Los Angeles, CA, USA) placed at the center of both the Peltier elements served as a visual cue, which has been shown to enhance attraction for warm surfaces in *Aedes* mosquitoes [12,14] (Figure 2A). At the beginning of each 75 min long trial, 15 female mosquitoes were released into the cage and allowed to acclimate for 5 min. During acclimation, both Peltier elements were maintained at ambient temperature (23 °C). Post acclimation, one of the Peltier elements (Peltier*_warm_*), chosen at random, was warmed up to deliver an increasing sequence of thermal stimuli between 30 and 50 °C with a 5 °C difference between successive stimuli, i.e., 30, 35, 40, 45, and 50 °C. The sequence of thermal stimuli in the assay was not randomized as exposure to higher temperatures in the initial phase of the assay might affect mosquitoes’ subsequent responses towards thermal stimuli at lower temperatures. Each of these thermal stimuli lasted for 10 min, following which the Peltier was cooled to ambient temperature for 5 min (Figure 2B). A humidified air stream (6.80 m/s) was delivered into the cage from the center of its top side throughout the duration of the assay. A CO_2_ pulse (2100 ppm or 30,000 ppm; 0.8 m/s, Gasco, Oldsmar, FL, USA) lasting 2 min accompanied the onset of each thermal stimulus and was injected into the humidified air circuit (Figure 2A). The other Peltier element (Peltier*_ambient_*) was maintained at ambient temperature throughout the trial. The surface temperatures were monitored using a thermal imaging camera (C3, FLIR Systems, Wilsonville, OR, USA) and precisely controlled via a custom-built Arduino PID controller (Arduino Uno R3; Monster Moto Shield VNH2SP30). The circuit diagram and code for the PID controller used in this assay are available online (https://github.com/mosquito-hub/Culex-Thermal-Biology.git, accessed on 10 January 2022). Gloves were used to release mosquitoes into the experimental setup to avoid contamination with host odors, and the experiment was triggered and controlled remotely to prevent interference from the experimenter. Mosquitoes were attracted to the Peltier surface as well as the adjacent surfaces, perhaps owing to heat dissipation and convective currents. To account for attraction elicited by the dissipated heat, a target region (9 × 9 cm) around each of the Peltier elements was defined within which the number of mosquitoes that landed every 30 s throughout the trial was quantified by manually transcribing the videos.

### 2.4. Statistical Analyses

#### 2.4.1. Thermopreference Assays

Data from the thermopreference assays were imported into R [42] for analysis and visualization. In a first step, the distribution of the temperature preferred by mosquitoes for each experiment and species was compared to a uniform, continuous distribution by means of Kolmogorov–Smirnov tests. Then, the effect of the species and experiments on the preferred temperatures and relative humidities were analyzed by means of Linear Models (LM) with the species (3 levels: *Cx. quinquefasciatus*, *Cx. tarsalis*, *Cx. territans*) and gradient types (3 levels: constant, low, high) as categorical fixed predictors and a gaussian error distribution. Tukey post hoc tests with *p* value adjustment were used as a follow-up analysis for multiple comparisons, using the R packages *lme4* (version 1.1–27.1 [43]), *multcomp* (version 1.4–17 [44]), and *emmeans* (version 1.7.1–1 [45]).

In our thermal preference assays, mosquitoes either landed on the substrate (i.e., the aluminum plate of the gradient) or the sidewalls of the acrylic covers. To compare the proportions of mosquitoes landing on either the substrate or the sidewalls of the apparatus we used a Generalized Linear Model with a binomial error distribution and a logit link. The species (3 levels: *Cx. quinquefasciatus, Cx. tarsalis, Cx. territans*) and gradient types (3 levels: constant, low, high) were used as categorical fixed predictors in the model. Post hoc pairwise comparisons between species and gradient types were achieved with the Tukey method for *p* value adjustment using the R packages *lme4* [43] and *emmeans* [45]. Visualization of the location of each mosquito landed either on the substrate or the sidewalls of the gradient was achieved by scaling the coordinates between 0 (the minimum recorded value) and 1 (the maximum recorded value) according to the formula:

$$Location_{scaled}=\frac{(Value_{i}-min\left(Value\right)}{(max\left(Value\right)-min\left(Value\right)},$$

where *i* represents each individual mosquito.

#### 2.4.2. CO_2_-Gated Heat Seeking Assays

Data from the heat-seeking assay were analyzed using Generalized Linear Mixed Models with a Penalized Quasi-Likelihood approach (*glmmPQL* in R package *MASS*, version 7.3–54 [46]). The model assumed binomially distributed errors with proportion of mosquito landings on Peltier*_warm_* as the response variable. The species of *Culex* mosquitoes (*Cx. quinquefasciatus*, *Cx. tarsalis*, *Cx. territans*), CO_2_ concentrations (2100 ppm and 30,000 ppm), and ambient (23 °C) vs. warm (30 °C to 50 °C) thermal stimuli are the categorical fixed predictor variables included as fixed effects in the model. The proportion of mosquito landings on Peltier*_ambient_* was included as a random effect. As this dataset involves repeated measurements on fifteen mosquitoes per assay across time points, to account for temporal correlations in the response variable, the residual correlation structure was incorporated in the model using Autoregressive order 1 (AR-1) function [47]. The random effect in the model represents variations in mosquito responses resulting from mere presence of the Peltier elements in the experiment cage in the absence of any thermal stimulus. A three-way interaction between the predictor variables was modeled to formally test for species-specific responses towards thermal stimuli set at ambient and host-like temperatures before and after CO_2_ exposure. Post-hoc analysis for significant effects was performed using Tukey’s HSD test and the reported *P* values are adjusted for multiple comparisons (Tukey’s method) using the function *emmeans* (in R package *emmeans*, version 1.7.1–1 [45]). In a subsequent analysis, the mosquito responses to the thermal stimuli (10 min per stimulus; 30–50 °C) were compared across five 2-min intervals. A CO_2_ pulse accompanied the first of the five 2-min intervals for every thermal stimulus. Finally, the proportion of mosquitoes landing on Peltier*_warm_* at 23 °C (5 min ahead of every thermal stimulus) was compared across thermal stimuli to test for the effects of multiple exposures to thermal stimuli on mosquito heat-seeking behavior. All results are presented as effect sizes with the corresponding 95% confidence intervals. Statistical significance was determined at an experiment-wise α = 0.05. We used R version 3.6.2 [42] to perform all the analyses and visualize the data (using R package *ggplot2*, version 3.3.5 [46]).

## 3. Results

### 3.1. Thermopreference Assays

**Figure 3:**
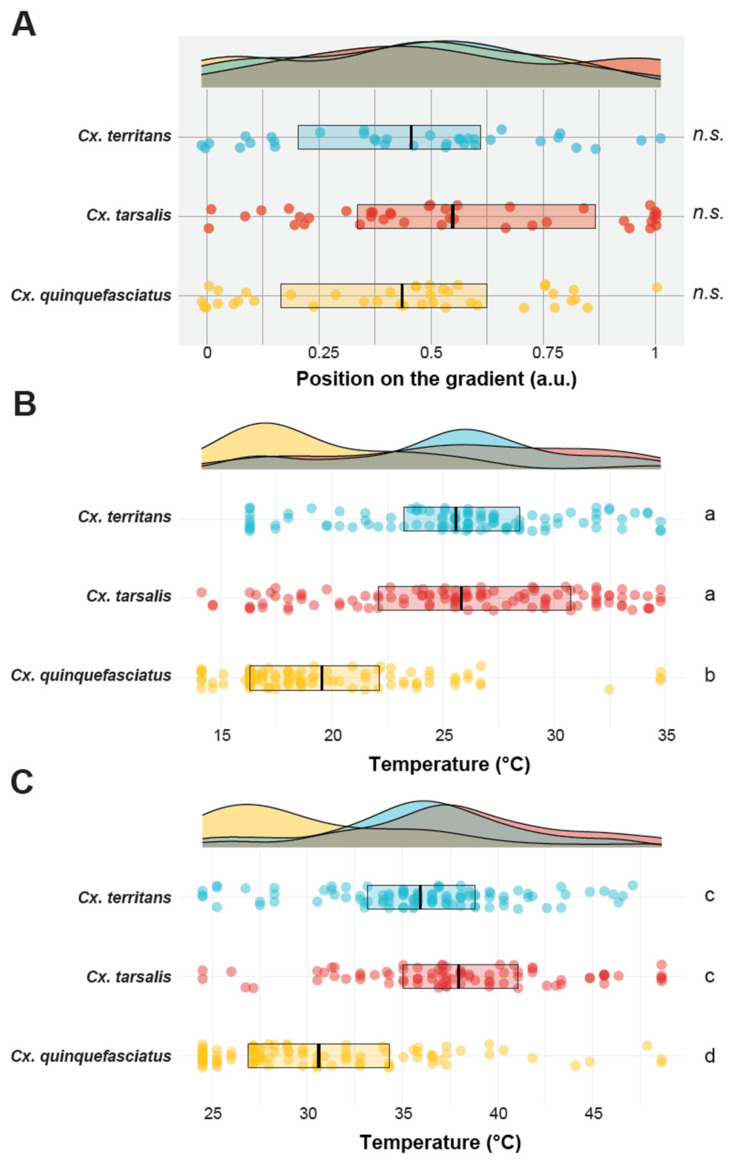
Thermopreference in *Culex* mosquitoes. (**A**). Mosquito distribution (bottom) on the aluminum plate set at 25 °C (i.e., constant temperature, control) and corresponding density plot (top). (**B**). Mosquito distribution (bottom) on the low temperature gradient (bottom) and corresponding density plot (top). (**C**). Mosquito distribution (bottom) on the high temperature gradient (bottom) and corresponding density plot (top). The density plots summarize the mosquitoes’ distribution along the gradient. Each dot represents the final resting position (i.e., T*_p_*) of a single female mosquito. The boxes represent the upper and lower quartiles, and the black bars indicate the mean of each group. *n.s.* denotes mosquito distributions in the constant gradient experiments that were not significantly different from a uniform, continuous distribution (Kolmogorov–Smirnov tests, α = 0.05). Letters denote statistical differences between groups (Tukey post hoc tests for multiple comparisons, adjusted α = 0.05). Four replicates (n = 10; N = 40) have been used for the constant gradient. Ten replicates (n = 10, N = 100) per species have been used for the low and high gradients.

All three mosquito species displayed even and continuous distribution when provided with a constant temperature (i.e., 25 °C), which indicated no spatial preference, thigmotaxis, or bias relating to the setup and environment (Kolmogorov–Smirnov tests: *p* = 0.42, *p* = 0.08, and *p* = 0.28 for *Cx. quinquefasciatus*, *Cx. tarsalis*, and *Cx. territans*, respectively) (Figure 3A). In the low gradient experiments, all species displayed a significant difference from a continuous distribution (Kolmogorov–Smirnov tests: *p* < 0.04). *Culex tarsalis* was more distributed throughout the aluminum plate than *Cx. territans,* which concentrated on the center of the gradient, yet they exhibited similar average T*_p_* (low: T*_p_* = 25.8 ± 5.6 °C and T*_p_* = 25.6 ± 4.9 °C, respectively; Tukey Contrasts for multiple comparisons of means: *p* = 0.9). Similarly, in the high gradient experiments, both species aggregated principally in the center of the gradient with *Cx. tarsalis* exhibiting a slightly higher average T*_p_* compared to *Cx. territans* (T*_p_* = 38 ± 5.6 °C and T*_p_* = 35.9 ± 5.2 °C, for *Cx. tarsalis* and *Cx. territans,* respectively) (Tukey Contrasts for multiple comparisons of means: *p* = 0.11) (Figure 3B). Although both *Cx. territans* and *Cx. tarsalis’* distributions were significantly different from continuous distributions, only *Cx. tarsalis’* was not different from a normal, gaussian, distribution (all Kolmogorov–Smirnov tests: *p* < 0.008; Shapiro–Wilk normality test: *p* = 0.037 and *p* = 0.113 for *Cx. territans* and *Cx. tarsalis*, respectively). *Cx. quinquefasciatus* behaved differently compared to *Cx. tarsalis* and *Cx. territans* in both low and high gradient experiments. Indeed, these mosquitoes showed a preference for the coolest spot available on the gradient (low: T*_p_* = 19.5 ± 4.6 °C and high: T*_p_* = 30.6 ± 5.9 °C), a behavior that was not observed during the control (i.e., constant temperature) experiment (Tukey Contrasts for multiple comparisons of means: *p* < 0.001 for all pairwise comparisons) (Figure 3A–C).

**Figure 4:**
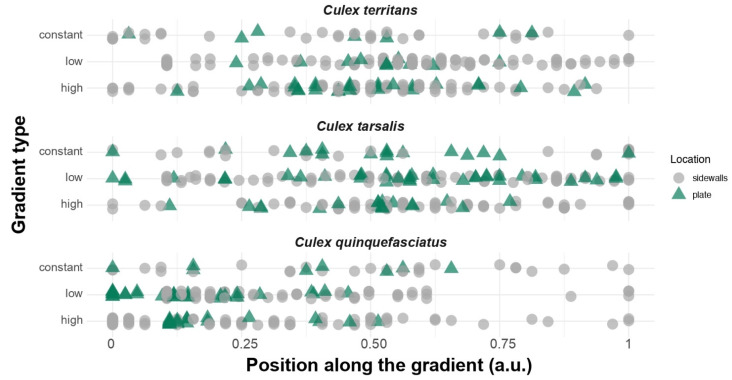
Proportion of mosquitoes resting on the aluminum plate (i.e., “plate”, green triangle) and on the side of the covers (i.e., “sidewalls”, grey circle) for each of the temperature gradients and mosquito species tested. Four replicates (n = 10; N = 40) have been used for the constant gradient. Ten replicates (n = 10; N = 100) per species have been used for the low and high gradients.

The proportion of mosquitoes resting on the sides of the covers versus on the plate was higher in *Cx. territans* (89.1 ± 3.3%) compared to both *Cx. tarsalis* (66.1 ± 5.6%) and *Cx. quinquefasciatus* (69.0 ± 5.6%) for the low gradient experiments (Pairwise comparisons on the log odds ratio scale with Tukey method for *p* value adjustment: *p* = 0.0057 and *p* = 0.0256, respectively) (Figure 4). However, the proportion was similar in the three species for the high gradient experiments (*Cx. quinquefasciatus*: 72.0 ± 5.3%; *Cx. tarsalis*: 75.6 ± 5.4%; *Cx. territans*: 70.8 ± 5.5%, respectively). (Pairwise comparisons on the log odds ratio scale with the Tukey method for *p* value adjustment: *p* > 0.99) (Figure 4). Interestingly, 20% of *Cx. tarsalis* mosquitoes were found knocked down on the warmer side of the gradient during the high gradient experiments, which did not occur in either of the other species.

**Figure 5:**
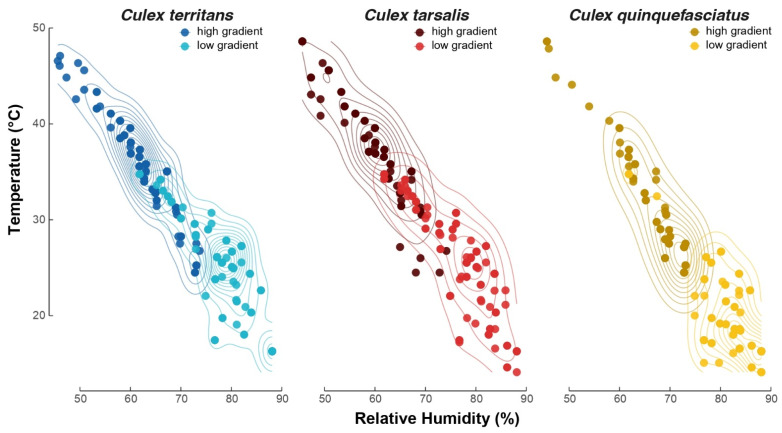
A 2-D density plot representing the temperature and humidity at the final resting position of the three *Culex* mosquito species on the gradients. Each dot represents a single mosquito. Low and high gradient data are highlighted in light and dark shades, respectively. Ten replicates (n = 10; N = 100) per condition per species have been used.

In these experiments, while the humidity gradient was minimized by the introduction of moist wipes, a humidity gradient, negatively correlated with the temperature gradient (Pearson’s product–moment correlation: *R*^2^ = −0.95; *p* < 0.001). Given this strong correlation, the hygric preference across gradients and species mirrors the patterns observed with the thermal preferences, where *Cx. quinquefasciatus* significantly preferred more humid locations on the gradients (82.2 ± 0.648% RH and 67.5 ± 0.648% RH for the low and high thermal gradients, respectively) than *Cx. tarsalis* (76.5 ± 0.621 and 59.2 ± 0.716% RH for the low and high thermal gradients, respectively) and *Cx. territans* (77.6 ± 0.645 and 61.8 ± 0.662% RH or the low and high thermal gradients, respectively). (Tukey post hoc tests: *p* < 0.001 for all comparisons). No significant differences were found between *Cx. tarsalis* and *Cx. territans* in the low (Tukey post hoc test: *p* = 0.788) and high gradients (Tukey post hoc test: *p* = 0.067), but within each species hygric preferences were significantly higher in the low than the high gradients (Tukey post hoc tests: *p* < 0.001 for all comparisons), reflecting the higher humidity levels correlated with lower temperatures (Figure 5).

### 3.2. CO_2_-Gated Heat Seeking Assays

**Figure 6:**
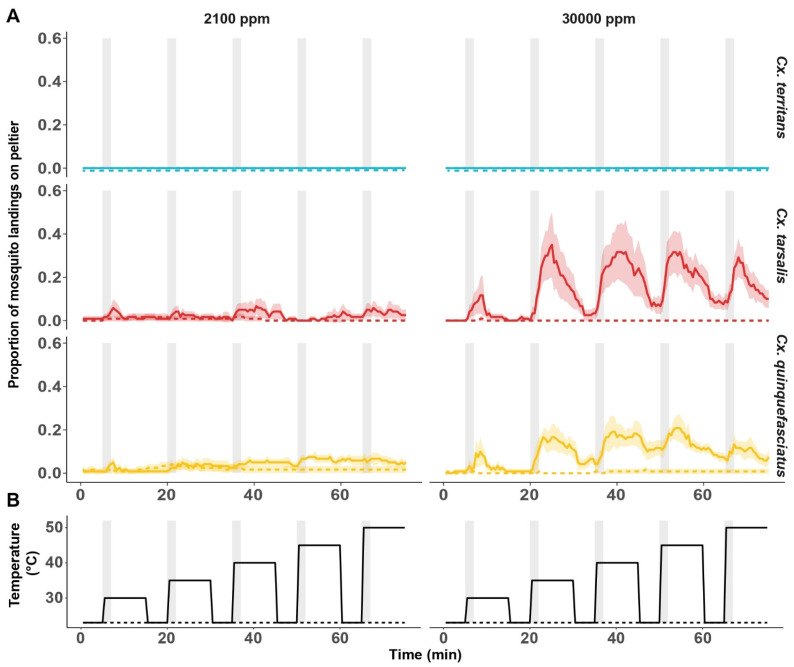
Heat seeking of *Culex* mosquitoes exposed to pulsed CO_2_ at low or high concentrations. (**A**). Mean proportion of mosquito landings on the ambient (23 °C; dashed line) and warm (30–50 °C; solid line) Peltier elements in the presence and absence of CO_2_ at concentrations of 2100 ppm (left) and 30,000 ppm (right). The shaded region around the mean denotes the 95% confidence interval. (**B**). Schematic representation of Peltier elements’ temperatures, both ambient (dashed line) and warm (solid line), as a function of time in the heat-seeking assay. Vertical gray bars denote the 2-min CO_2_ pulses that accompanied the thermal stimuli in the assay. Eight replicates (n = 15) per condition per species have been used.

*Culex territans* did not respond to any thermal stimuli both in the presence and absence of CO_2_ at both 2100 ppm and 30,000 ppm (Figure 6) (i.e., no mosquito landed on Peltier*_ambient_* and Peltier*_warm_*). Therefore, the responses of *Cx. territans* were excluded from subsequent analysis. The heat-seeking responses of both *Cx. tarsalis* and *Cx. quinquefasciatus* were contingent on exposure to CO_2_ (Table 1; Supplementary Tables S1 and S2). The response of both species towards Peltier*_warm_* (30–50 °C) was significantly higher after exposure to CO_2_ at 30,000 ppm when compared to 2100 ppm (Table 1; Supplementary Tables S1 and S2). Post exposure to CO_2_ at 30,000 ppm, the proportion of heat-seeking *Cx. tarsalis* and *Cx. quinquefasciatus* increased significantly with the temperature of Peltier*_warm_* and was the highest at 40 and 45 °C (Figure 6A; Table 1). Between the two species, post exposure to CO_2_ at 30,000 ppm, the magnitude of heat-seeking response of *Cx. tarsalis* towards Peltier*_warm_* (30–50 °C) was significantly higher when compared to *Cx. quinquefasciatus* (Chisq: 15.22, *p* < 0.01; Supplementary Tables S1 and S2). Post exposure to a lower concentration of CO_2_, i.e., 2100 ppm, significantly fewer *Cx. tarsalis* and *Cx. quinquefasciatus* responded to the thermal stimuli from Peltier*_warm_* set between 30 and 50 °C (Figure 6A; Table 1). Post exposure to 2100 ppm CO_2_, the heat-seeking responses of *Cx. tarsalis* and *Cx. quinquefasciatus* were not significantly different between 30 and 40 °C (Figure 6A; Table 1). However, the proportion of heat-seeking *Cx. quinquefasciatus* towards Peltier*_warm_* set at 45 and 50 °C upon exposure to 2100 ppm of CO_2_ was significantly more than *Cx. tarsalis* (Table 1; Supplementary Tables S1 and S2).

The heat-seeking response of both *Cx. tarsalis* and *Cx. quinquefasciatus* towards Peltier*_warm_* between 30 and 50 °C peaked at 2 min post exposure to CO_2_ (at 2100 and 30,000 ppm) and declined consistently thereafter over the remaining 6 min (Table 2; Supplementary Tables S3 and S4). While the activity of the mosquitoes was not quantified in this assay, *Cx. tarsalis* and *Cx. quinquefasciatus* were actively flying during their exposure to CO_2_ at 30,000 ppm. The magnitude of flight activity and the number of active mosquitoes in the two species were lower during exposure to 2100 ppm. *Culex territans* did not exhibit any flight activity during exposure to CO_2_ at 2100 and 30,000 ppm.

Finally, to account for the effects of the increasing sequence of thermal stimuli in the assay, we compared the responses of *Cx. tarsalis* and *Cx. quinquefasciatus* towards Peltier*_warm_* at 23 °C before every thermal stimulus (Figure 6). With every exposure to increasing thermal stimuli in Peltier*_warm_*, irrespective of the CO_2_ concentration, significantly fewer mosquitoes moved away when Peltier*_warm_* was cooled to ambient temperature, i.e., 23 °C (Chisq: 17.98, *p* < 0.01; Table 3; Supplementary Tables S5 and S6), which could be due to the time interval (5 min) between thermal stimuli presentations.

## 4. Discussion

In this study, we found differences in thermopreference across three species of *Culex* mosquitoes. *Culex quinquefasciatus* selected the cooler and more humid locations of the thermal gradient on both low and high temperature gradients, whereas *Cx. territans* and *Cx. tarsalis* displayed higher T*_p_*. However, the distribution of these two species was different between the two temperature ranges tested here. *Culex tarsalis* was more evenly distributed in the low gradient compared to the high gradient experiments, revealing a thermal *preferendum* for warmer ambient temperatures than those tested in the low thermal gradient. *Culex territans*, however, showed similar distribution patterns in the low and high gradients except that the coolest temperatures of the low gradient and the warmest temperatures of the high gradient tended to be avoided. Altogether, this suggests a preference for temperatures between 20 and 40 °C for this species.

A species’ preference for the cooler or warmer resting temperatures may be related to the abiotic conditions associated with their natural environment. *Culex quinquefasciatus* is found mostly at low-to-moderate elevations throughout the tropical, subtropical, and warm temperate regions of the world [48,49]. *Culex tarsalis* is distributed across most of the USA in the subtropical, temperate, and desert regions, with the exception of the East coast and Southern Canada [21]. In contrast, *Cx. territans* is widely distributed throughout the Northern hemisphere and found in subtropical, temperate, and subarctic regions of the US, Canada, and Europe [21,22]. In addition to temperatures, each species experiences different humidity conditions in its native habitat, which may affect their risk of desiccation. Maintaining water balance is indeed particularly critical for mosquitoes and several *Culex* species can suppress water loss under unfavorable conditions and during diapause by adjusting their metabolic rates, changing their cuticle composition and by synthesizing HSPs [49,50,51,52]. Anderson and Hardwood [53] found that wild populations of *Cx. tarsalis* that diapause for longer periods of time tend to be more resistant to both cold and desiccation. Rinehart et al. [52] showed the same under laboratory conditions with *Cx. pipiens* reared under diapausing and non-diapausing conditions.

Mosquito strains used in the present study originate from various regions of the world that greatly differ in terms of annual average temperatures, rainfall and elevation. These abiotic factors can lead to variations in both bionomics and genomics, which could explain the results obtained in the present study. The *Cx. quinquefasciatus* strain tested here derives from eggs collected in Johannesburg, South Africa, where annual temperatures are mild (max: 26 °C, min: 15 °C) with a long dry season (Source: NOAA). Here, we found that this species selected cooler resting temperatures in both thermal gradients, which also correlates with higher humidity levels. *Culex tarsalis* was collected in California (Yolo county, CA, USA) where temperatures can average 35 °C during the summer and where rainfall is relatively low (Source: NOAA). The warm and dry conditions this mosquito is accustomed to could have shaped its preference for warmer and less humid conditions (i.e., this strain is more adapted to hot and dry conditions). *Culex territans* was collected at Mountain Lake Biological Station (VA, USA), which is at high elevation with humid cold winters and mild summers (max: 29 °C, min: 17 °C in July) (Source: MLBS weather station). Our data show that this species had an intermediate T*_p_* compared to the two other species. As inter-populations differences in terms of thermotolerance, resistance to desiccation [7,50], and bionomics [49] have been highlighted in *Culex* spp., it would be interesting to conduct experiments with populations of the same species originating from other regions of the world to further examine the extent to which environmental conditions in the native habitat influence *Culex* mosquito T*_p_*.

In addition to climatic conditions in the native habitat influencing T*_p_*, we could also hypothesize that *Cx. quinquefasciatus*’ selection for a cooler and more humid environment might also be due to host density and availability. Indeed, temperature may also affect the hosts’ behavior (e.g., overall activity, sheltering). However, due to their ability to regulate their body temperature, endotherms, which *Cx. quinquefasciatus* feeds on, might be available at cooler temperatures. In contrast, in a cooler environment, ectotherms targeted by *Cx. territans* and to a lesser extent *Cx. tarsalis*, might not be active at all. As heat is an important host-seeking cue for mosquito species feeding on endotherms, a contrast between the temperatures of the environment and of the host is needed to trigger host-seeking as well as biting. Thus, by selecting a lower T*_p_*, *Cx. quinquefasciatus* might increase their chances of detecting the heat signature of a potential host available in the surrounding environment. Determining T*_p_* of other tropical species would be beneficial to determine if this is a typical preference for species in these regions.

Beyond influencing the sites that adult mosquitoes will choose for resting, the environmental temperature also affects their overall activity and host-seeking as well as blood-feeding behaviors [7,8,9,10]. In these contexts, thermosensation plays an important role at close range from the host as mosquitoes use the convective plumes generated by animals to guide their landing orientation before initiating blood-feeding [11,12,13,14,15,16]. In addition to thermal cues, mosquitoes use multiple host-specific olfactory, visual, and gustatory cues along with carbon dioxide plumes to identify and locate potential hosts for blood feeding [16,54,55], which allows for disease transmission. In *Aedes aegypti,* while responses to established convective plumes have been observed in choice assays [16], the addition of CO_2_ was necessary to elicit landings on warm surfaces (i.e., Peltier elements) whose temperatures were transiently increased to natural host temperatures [13]. In the present study, the heat-seeking experiments revealed that *Cx. tarsalis* and *Cx. quinquefasciatus* also required the presence of CO_2_ at elevated levels (30,000 ppm) to respond to an object warmer than the ambient temperature. These two species displayed comparable response profiles as a function of the CO_2_ concentration, whereas *Cx. territans* did not show any marked responses, even at ecologically relevant (i.e., corresponding to their preferred host’s) levels of CO_2_ [56]. As *Cx. quinquefasciatus* feeds mainly on endotherms [33], and *Cx. tarsalis* is opportunistic but prefers endotherms [25,26,27,28], the similarity in their responses was expected. However, we found that the proportion of landing of *Cx. quinquefasciatus* was reduced at 50 °C, which reflects both their host preference as well as their lower T*_p_* compared to *Cx. tarsalis*. However, the continued interest in the Peltier_warm_ around 50 °C in *Cx. tarsalis* may exemplify their opportunistic feeding nature as well as their higher T*_p_*. *Culex territans*’ lack of response to the heat stimuli, regardless of the CO_2_ concentration, also reflects their host preference, as amphibians have no thermal signature and expel low levels of CO_2_ [56].

Several factors could contribute to these differences, including inter-specific variations in anatomical and morphological structures associated with host seeking as well as differences in the expression of receptors implicated in host detection [57,58]. Interestingly while *Cx. quinquefasciatus* and *Cx. tarsalis* are closely related species, *Cx. territans* is more phylogenetically distant from them [59,60,61,62,63]. Mosquitoes sense many aspects of their environment, including odorants, CO_2_, and heat, through specific receptors on their antennae, maxillary palps, tarsi and wings [64,65,66,67,68,69]. At the antennae level, a pair of thermoreceptors, one cold-sensitive and the other warm-sensitive, is housed together in a sensilla at the tip of the antennae. McIver [70] described structures in the first segment of the antennae in several *Culex* species and *Ae. aegypti*, which were later identified to mediate thermoception in *Ae. aegypti* mosquitoes [71]. Similar thermoreceptors, later characterized as TRPA1, have been identified in other mosquito species such as *Anopheles gambiae* [72] and *Culex pipiens* [73]. TRPA1, however, is known to be involved in mediating heat avoidance while *Ir21a*, a member of the ionotropic receptor (IR) family, has been found to be the primary receptor responsible for heat-seeking in *An. gambiae* and possibly other mosquito species [55]. Morphological studies showed that *Cx. territans* have fewer CO_2_-sensitive sensilla on their maxillary palps than *Cx. tarsalis* and *Cx. pipiens* [74], which could explain why, in the present study, *Cx. territans* did not respond to the heat stimuli combined with CO_2_. This could also be due to the absence of other sensory cues. Indeed, this species is known to use phonotaxis (i.e., frog calls) to locate their hosts [75]. In addition, our recent work has evinced the use of odors for host detection and blood feeding (Reinhold et al., in preparation). Gustatory receptors genes (Gr) are expressed in sensory neurons in sensilla on the maxillary palps and three GRs have been identified as critical for CO_2_ sensing in several mosquito genera, including *Anopheles*, *Aedes* as well as *Culex* [76,77,78]. However, knowledge on receptors associated with host seeking, in particular for heat and CO_2_ detection, in the three *Culex* species we focused on in this study remains limited, and future studies will be necessary to unravel the physiological and molecular mechanisms underlying host seeking in these disease vector insects.

## 5. Conclusions

In this study, we determined the T*_p_* and CO_2_-gated heat-seeking behaviors of three *Culex* spp., *Cx. tarsalis*, *Cx. territans*, and *Cx. quinquefasciatus.* We found that both the environmental conditions in the habitat of origin and host preferences of these three species impacted their T*_p_* and host seeking behavior. Future experiments will further dive into better understanding the thermal biology of these species at the behavioral and genetic levels.

## Figures and Tables

**Table 1:** Mosquito landings on Peltier_warm_ in the heat-seeking assay.

Species	Temperature of Peltier_warm_	CO_2_ Conc. (ppm)	% Mosquito Landings	Proportion	SE	Lower CI	Upper CI	*p*
*Cx. tarsalis*	30 °C	2100	3.97	0.040	0.005	0.007	0.186	0.027
		30,000	9.71	0.097	0.008	0.033	0.252	0.026
	35 °C	2100	3.7	0.037	0.005	0.007	0.184	0.027
		30,000	12.26	0.123	0.009	0.047	0.282	0.026
	40 °C	2100	3.8	0.038	0.005	0.007	0.184	0.027
		30,000	12.15	0.122	0.009	0.047	0.280	0.026
	45 °C	2100	3.49	0.035	0.005	0.006	0.182	0.027
		30,000	12.07	0.121	0.009	0.046	0.280	0.026
	50 °C	2100	3.47	0.035	0.005	0.006	0.183	0.028
		30,000	11.72	0.117	0.009	0.044	0.276	0.026
*Cx. quinquefasciatus*	30 °C	2100	4.51	0.045	0.005	0.010	0.185	0.026
		30,000	7.52	0.075	0.007	0.023	0.219	0.025
	35 °C	2100	5	0.050	0.006	0.012	0.191	0.026
		30,000	8.51	0.085	0.007	0.028	0.231	0.025
	40 °C	2100	5.16	0.052	0.006	0.012	0.192	0.025
		30,000	8.55	0.086	0.007	0.028	0.232	0.025
	45 °C	2100	5.2	0.052	0.006	0.012	0.193	0.025
		30,000	8.56	0.086	0.007	0.028	0.232	0.025
	50 °C	2100	5.13	0.051	0.006	0.012	0.192	0.025
		30,000	8.13	0.081	0.007	0.026	0.226	0.025

**Table 2:** Proportion of mosquito landings on Peltier_warm_ as a function of the duration of exposure to the thermal stimuli in the heat-seeking assay.

Species	Duration (min)	CO_2_ Pulse	CO_2_ conc. (ppm)	% Mosquito Landings	Proportion	SE	Lower CI	Upper CI	*p*
*Cx. tarsalis*	0–2	Yes	2100	0.06	0.001	-	0.000	1.000	0.999
			30,000	10.72	0.107	0.009	0.034	0.290	0.029
	2–4	No	2100	2.80	0.028	0.004	0.004	0.182	0.029
			30,000	22.16	0.222	0.014	0.090	0.450	0.042
	4–6	No	2100	1.89	0.019	0.004	0.002	0.188	0.032
			30,000	17.86	0.179	0.014	0.062	0.415	0.039
	6–8	No	2100	2.23	0.022	0.003	0.003	0.147	0.027
			30,000	13.21	0.132	0.012	0.038	0.372	0.036
	8–10	No	2100	1.91	0.019	0.003	0.002	0.137	0.027
			30,000	9.61	0.096	0.011	0.020	0.355	0.037
*Cx. quinquefasciatus*	0–2	Yes	2100	3.72	0.037	0.005	0.008	0.165	0.025
			30,000	6.82	0.068	0.007	0.018	0.228	0.027
	2–4	No	2100	4.82	0.048	0.005	0.012	0.180	0.025
			30,000	13.37	0.134	0.010	0.048	0.322	0.030
	4–6	No	2100	3.76	0.038	0.005	0.007	0.176	0.026
			30,000	11.56	0.116	0.010	0.038	0.303	0.030
	6–8	No	2100	3.50	0.035	0.005	0.006	0.186	0.028
			30,000	8.97	0.090	0.009	0.025	0.272	0.029
	8–10	No	2100	3.36	0.034	0.005	0.005	0.181	0.028
			30,000	7.48	0.075	0.008	0.018	0.261	0.029

**Table 3:** Proportion of mosquito landings on Peltier_warm_ at 23 °C before every thermal stimulus in the heat-seeking assay.

Species	Peltier_warm_ at 23 °C	CO_2_ Conc. (ppm)	% Mosquito Landings	Probability	SE	Lower CI	Upper CI	*p*
*Cx. tarsalis*	before 30 °C	2100	0.83	0.008	0.002	0.001	0.120	0.029
		30,000	0.00	0.000	0.000	0.000	1.000	1.000
	before 35 °C	2100	1.17	0.012	0.002	0.001	0.111	0.027
		30,000	0.50	0.005	0.001	0.000	0.154	0.034
	before 40 °C	2100	1.17	0.012	0.002	0.001	0.111	0.027
		30,000	4.92	0.049	0.004	0.016	0.143	0.020
	before 45 °C	2100	1.50	0.015	0.002	0.002	0.109	0.025
		30,000	11.67	0.117	0.006	0.057	0.225	0.020
	before 50 °C	2100	2.25	0.023	0.003	0.004	0.113	0.023
		30,000	10.33	0.103	0.006	0.048	0.209	0.019
*Cx. quinquefasciatus*	before 30 °C	2100	0.92	0.009	0.002	0.001	0.117	0.028
		30,000	0.58	0.006	0.002	0.000	0.141	0.032
	before 35 °C	2100	0.83	0.008	0.002	0.001	0.120	0.029
		30,000	0.83	0.008	0.002	0.001	0.120	0.029
	before 40 °C	2100	3.17	0.032	0.003	0.008	0.122	0.021
		30,000	5.58	0.056	0.005	0.019	0.151	0.020
	before 45 °C	2100	4.42	0.044	0.004	0.013	0.137	0.020
		30,000	11.92	0.119	0.006	0.058	0.228	0.020
	before 50 °C	2100	6.08	0.061	0.005	0.022	0.158	0.019
		30,000	8.83	0.088	0.006	0.038	0.191	0.019

## Data Availability

Data can be accessed online https://github.com/mosquito-hub/Culex-Thermal-Biology.git (accessed on 10 January 2022) and upon email request.

## Supplementary Materials

The following are available online at https://www.mdpi.com/article/10.3390/insects13010092/s1, Table S1: Analysis of deviance table for the Generalized Linear Mixed Model analyzing the proportion of mosquito landings on Peltier_warm_ in the heat seeking assay (data in Table 1), Table S2: Pairwise contrasts for the proportion of mosquito landings on Peltier_warm_ in the heat seeking assay (data in Table 1), Table S3: Analysis of deviance table for the Generalized Linear Mixed Model analyzing the proportion of mosquito landings on Peltier_warm_ as a function of the duration of exposure to the thermal stimuli in the heat seeking assay (data in Table 2), Table S4: Pairwise contrasts for the proportion of mosquito landings on Peltier_warm_ as a function of the duration of exposure to the thermal stimuli in the heat seeking assay (data in Table 2), Table S5: Analysis of deviance table for the Generalized Linear Mixed Model analyzing the proportion of mosquito landings on Peltier_warm_ at 23°C before every thermal stimulus in the heat seeking assay (data in Table 3), Table S6: Pairwise contrasts for the proportion of mosquito landings on Peltier_warm_ at 23 °C before every thermal stimulus in the heat seeking assay (data in Table 3).
