# Supplementary material for: Species-Specificity in Thermopreference and CO2-Gated Heat-Seeking in Culex Mosquitoes
Source: Insects. 2022 Jan 14;13(1):92. doi: 10.3390/insects13010092 (PMC8779787; doi:10.3390/insects13010092)
Supplement: Supplementary file 1 [file insects-13-00092-s001.zip › insects-1536378-supplementary.pdf]

Supplementary Table S1: Analysis of deviance table for the Generalized Linear Mixed Model analyzing the proportion of mosquito landings on $Peltier_{warm}$ in the heat seeking assay (data in Table 1)

	Chisq	Df	*P* (>Chisq)
Species	3.61	1	0.057
CO_2 conc.	74.41	1	< 0.001
Temp($Peltier_{warm}$)	43.51	5	< 0.001
Species : CO_2 conc.	12.97	1	< 0.001
Species : Temp($Peltier_{warm}$)	0.95	5	0.967
CO_2 conc. : Temp($Peltier_{warm}$)	12.96	5	< 0.05
Species : CO_2 conc. : Temp($Peltier_{warm}$)	15.22	5	< 0.01

Supplementary Table S2: Pairwise contrasts for the proportion of mosquito landings on $Peltier_{warm}$ in the heat seeking assay (data in Table 1)

Contrast	Temperature of $Peltier_{warm}$	CO_2 conc. (ppm)	Odds ratio	Lower CI	Upper CI	*P*
Cx. tarsalis - Cx. quinquefasciatus	30	2100	0.876	0.623	1.231	0.4452
		30000	1.322	1.03	1.698	0.0285
	35	2100	0.731	0.519	1.028	0.0719
		30000	1.501	1.19	1.894	0.0006
	40	2100	0.727	0.519	1.018	0.0631

		30000	1.479	1.173	1.866	0.0009
	45	2100	0.66	0.468	0.931	0.0178
		30000	1.466	1.162	1.85	0.0012
	50	2100	0.664	0.47	0.939	0.0206
		30000	1.501	1.185	1.901	0.0008

Supplementary Table S3: Analysis of deviance table for the Generalized Linear Mixed Model analyzing the proportion of mosquito landings on Peltier$_{warm}$ as a function of the duration of exposure to the thermal stimuli in the heat seeking assay (data in Table 2).

	**Chisq**	**Df**	***P* (>Chisq)**
Species	214.87	10	< 0.001
CO_2 conc.	1759.33	10	< 0.001
Temp(Peltier$_{warm}$)	406.00	23	< 0.001
Stim. duration	259.73	19	< 0.001
Species : CO_2 conc.	231.82	5	< 0.001
Species : Temp(Peltier$_{warm}$)	31.23	13	< 0.01
CO_2 conc. : Temp(Peltier$_{warm}$)	79.49	13	< 0.001
Species : Stim. duration	17.82	11	0.086
CO_2 conc. : Stim. duration	44.84	11	< 0.001

Temp(Peltier$_{warm}$) : Stim. duration	114.37	31	< 0.001
Species : CO_2 conc. : Temp(Peltier$_{warm}$)	39.39	8	< 0.001
Species : CO_2 conc. : Stim. duration	5.55	7	0.593
Species : Temp(Peltier$_{warm}$) : Stim. duration	18.23	23	0.745
CO_2 conc. : Temp(Peltier$_{warm}$) : Stim. duration	15.77	23	0.865
Species : CO_2 conc. : Temp(Peltier$_{warm}$) : Stim.duration	11.71	20	0.926

Supplementary Table S4: Pairwise contrasts for the proportion of mosquito landings on Peltier$_{warm}$ as a function of the duration of exposure to the thermal stimuli in the heat seeking assay (data in Table 2).

Contrast	Duration (min)	CO_2 pulse	CO_2 conc. (ppm)	Odds ratio	Lower CI	Upper CI	*P*
Cx. tarsalis - Cx. quinquefasciatus	0 - 2	Yes	2100	0.0146	0	–	0.9993
			30000	1.6385	1.313	2	<.0001
	2 - 4	No	2100	0.5685	0.402	1	0.0014
			30000	1.8445	1.58	2	<.0001
	4 - 6	No	2100	0.4937	0.321	1	0.0013
			30000	1.663	1.379	2	<.0001
	6 - 8	No	2100	0.6273	0.427	1	0.0176
			30000	1.5446	1.226	2	0.0002

	8 - 10	No	2100	0.5594	0.377	1	0.004
			30000	1.3156	0.986	2	0.0627

Supplementary Table S5: Analysis of deviance table for the Generalized Linear Mixed Model analyzing the proportion of mosquito landings on Peltier$_{warm}$ at 23°C before every thermal stimulus in the heat seeking assay (data in Table 3).

	**Chisq**	**Df**	***P* (>Chisq)**
Species	107.25	9	< 0.001
CO_2 conc.	447.46	9	< 0.001
Before.temp	643.44	15	< 0.001
Species : CO_2 conc.	85.72	4	< 0.001
Species : Before.temp	33.65	7	< 0.001
CO2 conc. : Before.temp	97.41	7	< 0.001
Species : CO2 conc. : Before.temp	17.98	4	< 0.01

Supplementary Table S6: Pairwise contrasts for the proportion of mosquito landings on Peltier$_{warm}$ at 23°C before every thermal stimulus in the heat seeking assay (data in Table 3).

**Contrast**	**Peltier$_{warm}$ at 23°C**	**CO_2 pulse**	**CO_2 conc. (ppm)**	**Odds ratio**	**Lower CI**	**Upper CI**	***P***
Cx. tarsalis - Cx. quinquefasciatus	before 30°C	Yes	2100	0.908	0.501	2	0.7511
			30000	0	0	-	0.9996

	before 35°C	No	2100	1.405	0.8	2	0.2371
			30000	0.598	0.296	1	0.1509
	before 40°C	No	2100	0.361	0.235	1	<.0001
			30000	0.874	0.682	1	0.2896
	before 45°C	No	2100	0.33	0.227	0	<.0001
			30000	0.976	0.822	1	0.7834
	before 50°C	No	2100	0.355	0.261	0	<.0001
			30000	1.189	0.985	1	0.0711
